# Screen-Printed Electrodes: Promising Paper and Wearable Transducers for (Bio)Sensing

**DOI:** 10.3390/bios10070076

**Published:** 2020-07-09

**Authors:** Paloma Yáñez-Sedeño, Susana Campuzano, José Manuel Pingarrón

**Affiliations:** Departamento de Química Analítica, Facultad de CC. Químicas, Universidad Complutense de Madrid, E-28040 Madrid, Spain; susanacr@quim.ucm.es (S.C.); pingarro@quim.ucm.es (J.M.P.)

**Keywords:** screen-printed, electrochemical (bio)sensing, paper, wearable, environmental monitoring, clinical analysis

## Abstract

Screen-printing technology has revolutionized many fields, including that of electrochemical biosensing. Due to their current relevance, this review, unlike other papers, discusses the relevant aspects of electrochemical biosensors manufactured using this technology in connection to both paper substrates and wearable formats. The main trends, advances, and opportunities provided by these types of devices, with particular attention to the environmental and biomedical fields, are addressed along with illustrative fundamentals and applications of selected representative approaches from the recent literature. The main challenges and future directions to tackle in this research area are also pointed out.

## 1. Screen-Printed Paper Electrodes for (Bio)Sensing

Electrochemical paper-based analytical devices (e-PADs) combine the inherent advantages of electrochemical detection—such as high sensitivity and low detection limits (LODs), the possibility of enhancing selectivity by applying different potential values or using modified electrodes, and low cos—with those of paper—such as porosity, allowing liquid transport by capillarity, high surface area/volume ratio, and easy waste disposal by incineration [1]. Paper-based platforms are interesting alternatives to develop disposable, eco-friendly, and inexpensive electrochemical sensors. Lightness and flexibility are additional characteristics of these sensors, which confer unique exploitable properties for application in electroanalysis. Since 2009, when Dungchai et al. introduced e-PADs [2], research in this field has been intensive. Different fabrication procedures [3,4], materials [5], and various practical aspects [6,7,8] have been reviewed. Akyazi et al. [9] reported a critical overview on the fabrication techniques, production limitations, and the commercialization of paper devices.

In addition to general reports, a variety of methods using different configurations of (bio)sensors and paper-based microfluidic designs as detection platforms have been proposed. With the aim of providing comprehensive information, Table 1 and Table 2 summarize the fundamentals and main characteristics of relevant electroanalytical methods involving screen-printed paper-based devices applied to environmental and clinical monitoring, respectively.

## 2. Screen-Printed Paper Electrochemical (Bio)Sensors

### 2.1. Environmental Applications

An area in which screen-printed paper electrodes have shown particular relevance is environmental monitoring (Table 1). Different types of paper impregnated with suitable reagents [10], modified with metal nanoparticles or carbon nanostructures both in the absence or in the presence [11] of specific enzymes, have been used to determine contaminants such as heavy metals [12], anions [13], and gases [14,15]. For instance, the electrocatalytic activity of polyoxymetalates (POMs) toward the electrochemical reduction of chlorate was employed to prepare a vanadium-containing POM ([PMO_11_VO_40_]^5−^) for the determination of ClO_3_^−^ in soils. Just like in other paper-based designs, the SPCE brought the electrodes and the paper together to create the appropriate cell volume for the electrolyte solution, resulting in a thin layer cell for electrochemical detection [10]. Moreover, in this particular application, paper also acted as a filter for soil analysis. Using chronocoulometry, a LOD of 0.31 mg mL^−1^ ClO_3_^−^ was achieved. Using a similar configuration, a disposable gas-sensing paper-based device (gPAD) was fabricated in origami design, integrating in a single device activated carbon as the gas adsorbent and the electrochemical detection consisting of a screen-printed graphene electrode modified with copper nanoparticles. Both NO and NO_2_ (as NOx) were detected with the same current responses measured by differential pulse voltammetry (DPV) achieving LODs of 0.23 vppm and 0.03 vppm with exposure times of 25 min and 1 h, respectively. Relative standard deviation (RSD) values less than 5.1% (n = 7 devices) for 25, 75, and 125 vppm NO_2_ were reported, and the gPAD was applied to detect NOx in air and exhaust gases from cars [14].

**Figure 1:**
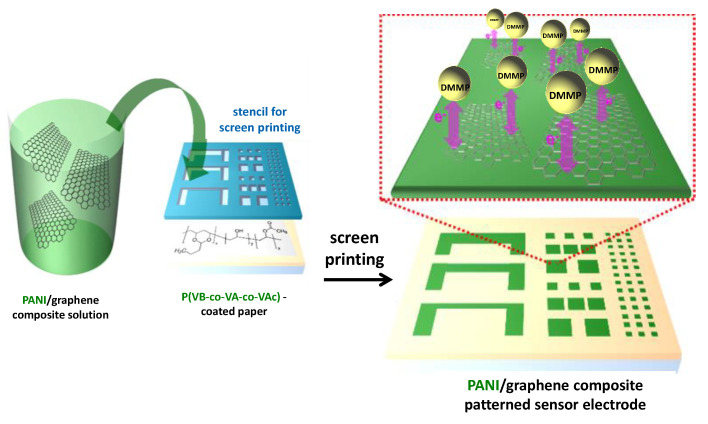
Fabrication process of conductive paper containing sensor patterns for dimethyl methylphosphonate (DMMP) based on poly(aniline) (PANI)/graphene composite. Reproduced and adapted with permission of American Chemical Society [15].

The nerve agent VX gas is prohibited as a chemical warfare agent. Since it cannot be used in research experiments, dimethyl methylphosphonate (DMMP) is utilized as a model. A conductive paper prepared with poly(aniline) (PANI) nanofiber and graphene sheet was used to detect DMMP at parts per billion within few seconds. The intermolecular forces between the cellulosic paper and the conductive additives were improved by using the copolymer poly(vinylbutyral-co-vinyl-alcohol-co-vinyl acetate) (Figure 1). The resulting sensor exhibited a detectable level of 3 ppb and a response time of 2 s [15]. In this field, strategies consisting of electrodes screen-printed onto a filter paper support allows enzymes and other reagents to be pre-loaded into the cellulose network. An illustrative example is the paper-based wearable electrode constructed for the detection of mustard agent, one of the most dangerous chemical warfare agents (CWAs). It is an origami-like device where the detection is based on the inhibitory effects of the analyte toward the enzyme choline oxidase. The amperometric responses were measured at a carbon black/Prussian blue nanocomposite distributed on the electrode surface and profiting its electrocatalytic activity for H_2_O_2_ reduction. A Keithley 2400 current source meter (Keithley Co., Cleveland, OH, USA) was used to measure the electrical properties and sensing performance of the conductive papers. The LOD attained in the aerosol phase was 0.019 g min m^−3^ [11].

A carbon-nanotubes-based ink prepared with sodium dodecyl sulfate (SDS) and chitosan (CS) absorbed onto cellulose fibers was used to prepare a paper electrode for the determination of Pb^2+^ trace levels in water samples. Square-wave anodic stripping voltammetry (SWASV) using a bismuth film prepared by in situ plating of Bi into the CNT-CS-SDS paper electrodes made the determination of Pb^2+^ in the presence of Bi (10–200 ppb) with a LOD of 6.74 ppb possible [12]. Furthermore, a paper-based, disposable electrochemical platform was developed for the determination of nitrite. Graphene nanosheets and gold nanoparticles were assembled to form a three-dimensional structure onto mixed cellulose ester (MCE) filter papers, leading to thin layer rather than planar diffusion behavior of nitrite at the paper-based electrode. The resulting platform provided larger currents compared to conventional gold or glassy carbon electrodes and, consequently, allowed an improved sensitivity. Importantly, this design effectively avoided the fouling arising from the adsorption of oxidation products thus allowing the determination of nitrite in environmental samples such as waters and industrial sewage. The calibration curve at +0.74 V covered a wide concentration range of 0.3–720 μM, and the LOD was 0.1 μM (S/N = 3) [13].

Contamination of water by microbial pathogens leading to water-borne diseases requires strict controls in drinking water resources, particularly in poor regions, to reduce mortality incidence. Although not included in Table 1, various methods related to the detection of microorganisms should be highlighted. For instance, a simple low-cost paper-based impedimetric sensor for the detection of bacteria in water was prepared using carbon electrodes screen-printed with a conductive ink onto a commercial hydrophobic paper. Concanavalin A covalently immobilized onto the carboxylated electrode surface was used as the biorecognition element due to its ability to selectively interact with mono- and oligo-saccharides on bacteria. In this method, the hydrophobicity of the cellulose paper used as a substrate prevented any unspecific adsorption. The biosensor was applied to bacterial cultures from sewage sludge that were grown in synthetic water, then filtered and enumerated for defining the stock solution. The calibration plot showed an increase in the charge transfer resistance (R_CT_) over the 10^3^ to 10^6^ colony forming unit (CFU) mL^−1^ range, with an estimated LOD of 1.9×10^3^ CFU mL^−1^ [16]. A fast-flow paper-based electrochemical sensor was developed by Channon et al. for the label-free detection of virus particles [17]. West Nile viruses were detected by electrochemical impedance spectroscopy using antibody functionalized Au microwires, achieving a LOD of 10.2 particles in 50 μL of cell culture media. The sensing approach is easily controllable by means of a smartphone and may presumably be applied to a range of biological targets. Toxins secreted from pathogens can also be detected in bacterial cultures using paper-based electrochemical sensors. This is the case of pyocyanin, a toxin solely produced by *Pseudomonas aeruginosa*, whose detection was performed using an inexpensive approach involving electrode printing of carbon ink on photo paper and square wave voltammetry [18]. It is worth noting that some authors have also exploited the bacterial enzyme activity (expression of β-glucosidase by *Enterococcus* spp. and the production of β-galactosidase and β-glucuronidase by *E. coli*) for their determination at paper-based electrochemical sensors [19]. 

The use of biological indicators to determine the toxicological effect of environment pollutants is an interesting research area where microorganisms combined with paper-based electrochemical sensors provide important advantages. In this context, a μPAD for highly integrated biotoxicity measurements was prepared involving screen-printing with conductive carbon ink and chromatographic paper. The μPAD contained three functional units for injection, separation, and detection zones with hydrophobic barriers. The *E. coli* cell incubation and the fluid-cell separation were integrated as special microfluidic units, and an interesting scheme for determination making use of inhibition of the microorganism respiratory chain was utilized. The procedure involved the addition of benzoquinone (BQ) to react with the electron or hydrogen carriers including enzymes, co-enzymes, prosthetic groups, or co-factors, which compose the respiratory chain, to form hydroquinone (HQ). When toxic environment inhibits the cellular respiratory chain, the quantity of HQ decreases leading to lower electrochemical current. The relationship between toxicant and HQ production provided the fundamentals for the biotoxicity assay. As proofs of concept; two heavy metals, Cu^2+^ and Pb^2+^ in water and soil; the antibiotic penicillin in soil; and the pesticides acetamiprid, triazolone, and acephate in vegetable juices were detected [20].

### 2.2. Clinical Applications

Sensitive and selective sensors constructed with screen-printed electrode (SPEs) have been developed for different analytes of clinical relevance. These sensors exhibit great advantages allowing fabrication of attractive designs for single and multiple determination even in the absence of biological elements. In this context, the special features of paper as support material for the preparation of diagnostic devices, together with those of screen-printed platforms, represents an important advance for easy self-testing and point-of-care (POC) assessment. Table 2 summarizes the analytical characteristics and the main properties of some recent and representative methods applied to analytes of clinical interest in biological samples [27,28,29,30,31,32,33,34,35,36,37,38,39,40,41,42,43,44,45,46,47,48,49,50,51,52,53,54,55,56,57,58,59,60]. Some selected examples are discussed below. 

An illustrative example is a wax-printed paper-based device reported by Martins et al. [27] for the electrochemical detection of 3-nitrotyrosine (3-NT), a biomarker of oxidative stress. The paper was modified to become a hydrophobic support, and then carbon and silver conductive inks were applied to generate a three electrode-system on a small spot. Square wave voltammetry (SWV) was employed to determine 3-NT in a range from 500 nM to 1 mM with a low LOD of 49.2 nM. More recently, a disposable paper-based printed electroanalytical strip has been reported for the rapid and high-throughput detection of glutathione in blood [28]. The detection involved a thiol-disulfide exchange reaction giving an electroactive product easily oxidizable at a Prussian Blue/carbon black nanocomposite screen-printed onto a wax-patterned filter paper. The resulting configuration, where the paper provides a reagents-free device, allowed the detection of glutathione up to a concentration 10 mM, with a LOD value of 60 μM, and was employed to quantify blood glutathione at physiological levels.

**Figure 2:**
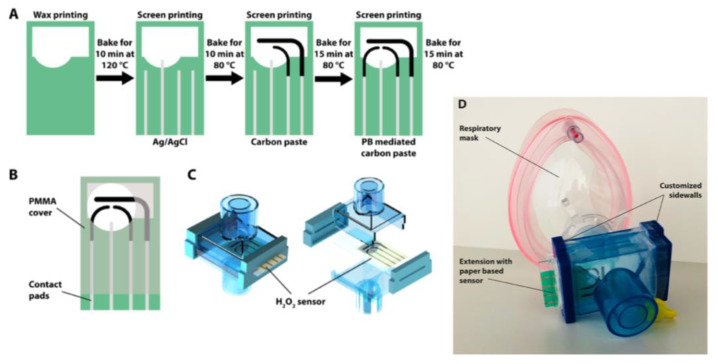
(**A**) Schematics of fabrication steps and (**B**) computer-aided design (CAD) drawing of the disposable cellulose paper-based electrochemical sensor for on-site testing of H_2_O_2_ in exhaled breath with poly-methylmethacrylate (PMMA) carrier. (**C**) Model of a filter extension for respiratory mask. (**D**) Image of respiratory mask with the commercial filter extension with customized sidewalls, containing the sensor chip. Reproduced and adapted with permission of American Chemical Society [29].

Hydrogen peroxide is an important biomarker associated with respiratory and pulmonary diseases such as asthma and lung cancer. A disposable cellulose paper-based electrochemical sensor integrated into a commercial respiratory mask was reported for on-site testing of H_2_O_2_ in exhaled breath (Figure 2) [29]. The device involved a Prussian-Blue-mediated carbon electrode for H_2_O_2_ detection and a carbon blank electrode for subtracting the background currents. In the presence of the analyte, the oxidation product formed from Prussian Blue was electrochemically reduced providing amperometric responses related to the H_2_O_2_ concentration. This configuration did not exhibit influence from environmental conditions or interferents due to differential measurements. In addition, the use of paper as flexible substrate and hygroscopic porous support eliminated the need for additional membranes.

**Figure 3:**
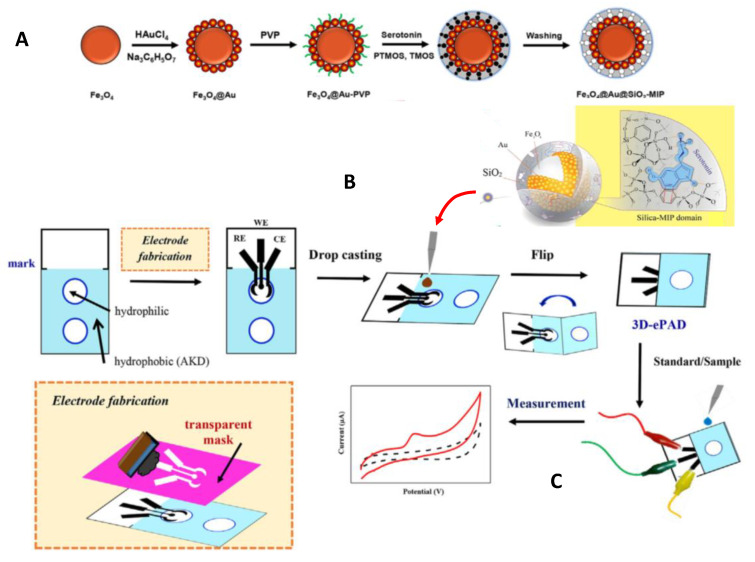
(**A**) Synthesis of Fe_3_O_4_@Au@SiO_2_-MIP, (**B**) preparation of the sensor for serotonin, and (**C**) electrochemical detection using the 3D-ePAD. Reproduced and adapted with permission of Elsevier [30].

Molecularly imprinted polymers (MIPs) used as electrode surface modifiers allow high selective recognition, although they sometimes lack the required sensitivity due to the poor conductivity of building materials. To improve the analytical performance, nanostructured configurations yielding larger currents and fast responses have been proposed. The resulting nano-sized MIPs have been combined with paper-based analytical devices to obtain three-dimensional electrochemical PADs (3D-ePADs), which provide additional advantages such as lower cost and smaller sample and reagents volumes. An interesting example is the method involving filter papers prepared by alkyl ketene dimer (AKD)-inkjet printing of a circular hydrophobic detection zone coupled with screen-printed graphite electrodes drop coated with Fe_3_O_4_@Au@SiO_2_-MIP nanocomposites (Figure 3), for the voltammetric determination of serotonin. Linear sweep voltammetry at +0.39 V provided a linear range from 0.01 to 1,000 mM with a LOD of 0.002 mM. The resulting MIPs exhibited strong affinity for the analyte, and the electrochemical sensor showed electrocatalytic activity toward the oxidation of serotonin. The sensor was successfully applied to the analysis of pharmaceutical capsules and urine samples [30].

**Figure 4:**
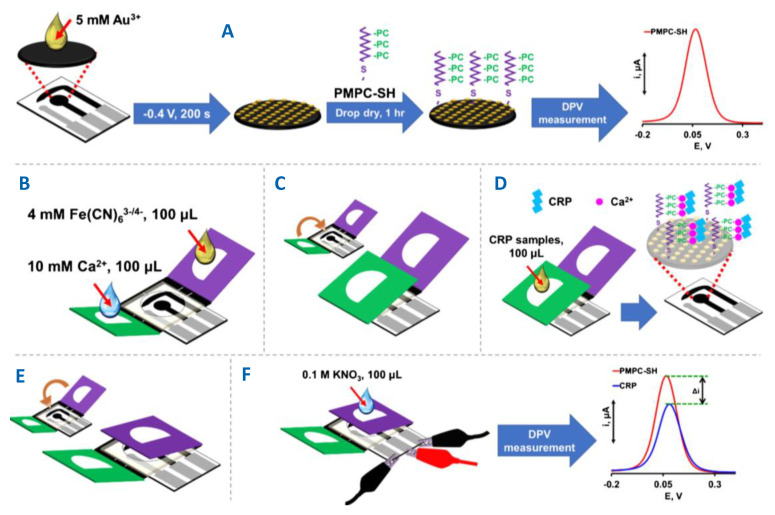
(**A**) Preparation of thiol-terminated poly(2-methacryloyloxyethyl phosphorylcholine) (PMPC-SH)-AuNPs/SPCE. (**B**–**F**) Steps for preparation of the PMPC-SH-AuNPs/SPCE/PAD sensor for the differential pulse voltammetry (DPV) determination of C-reactive protein (CRP). Reproduced and adapted with permission of Springer [31].

Human C-reactive protein (CRP) is a nonspecific pentameric protein produced by hepatocytes in the liver upon stimulation by endogenous proinflammatory cytokines. CRP is an important biomarker for various cardiovascular diseases and its determination requires sensitive and accurate methods with high selectivity for application in complex clinical samples. Pinyorospathum et al. [31] developed a single step method for the determination of CRP in human serum involving a AuNP-modified SPCE self- assembled with PADs tethered with a biomimetic polymer consisting of thiol-terminated poly(2-methacryloyloxyethyl phosphorylcholine) (PMPC-SH). The approach took advantage of the specific binding of protomers subunits forming the CRP structure to the phosphorylcholine group in the presence of calcium ion [32]. Figure 4A shows that PMPC-SH copolymer reacts with AuNPs; then, [Fe(CN)_6_]^3−/4−^ current at the resulting PMPC-SH/AuNPs-SPCE is measured by DPV to further subtracting it from the response in the presence of CRP. Figure 4B–F shows the preparation of the PAD in three parts—the middle where SPCE is placed, the green flap for the storage of calcium ions and dropping the sample, and the purple flap used for the detection with the [Fe(CN)_6_]^3−/4−^ redox probe. The current decreased in the presence of CRP and Ca^2+^ over the 5 to 5,000 ng mL^−1^ CRP concentration range with a LOD value of 1.6 ng mL^−1^. The use of a PMPC-modified surface reduced the nonspecific adsorption of proteins, and the sensor response was not interfered by bilirubin, myoglobin, or albumin. The sensor was successfully applied to the determination of CRP in certified human serum.

Electrochemical enzyme paper biosensors (EPADs) have been shown to provide an adequate microenvironment for direct measurements, while physical adsorption of the enzyme did not affect its native structure, function, and electrocatalytic activity [32]. An illustrative example is a biosensor for phenylketonuria (PKU) screening based on the determination of phenylalanine (Phe). The biosensor was implemented by immobilization of phenylalanine dehydrogenase (PheDH) over paper microzones placed onto an electrochemically reduced graphene oxide (ERGO)-modified SPCE. The detection of the NADH formed in the presence of NAD^+^ provided a sensitive, low-cost, and fast method for PKU monitoring in neonatal blood samples [33]. An original paper-based biosensor was developed for the detection of acetylcholinesterase (AChE) [34]. This enzyme catalyzes the hydrolysis of acetylcholine neurotransmitter and its abnormal function can promote and accelerate the aggregation of amyloid-betapeptides closely related to Alzheimer’s disease. The bioelectrode was fabricated by immobilization of acetylthiocholine on a sheet prepared with double adhesive tape. The enzyme samples were dropped on the backside of the electrode where, after hydrolysis, amperometric detection was performed and provided a LOD value of 0.1 U mL^−1^ AChE.

**Figure 5:**
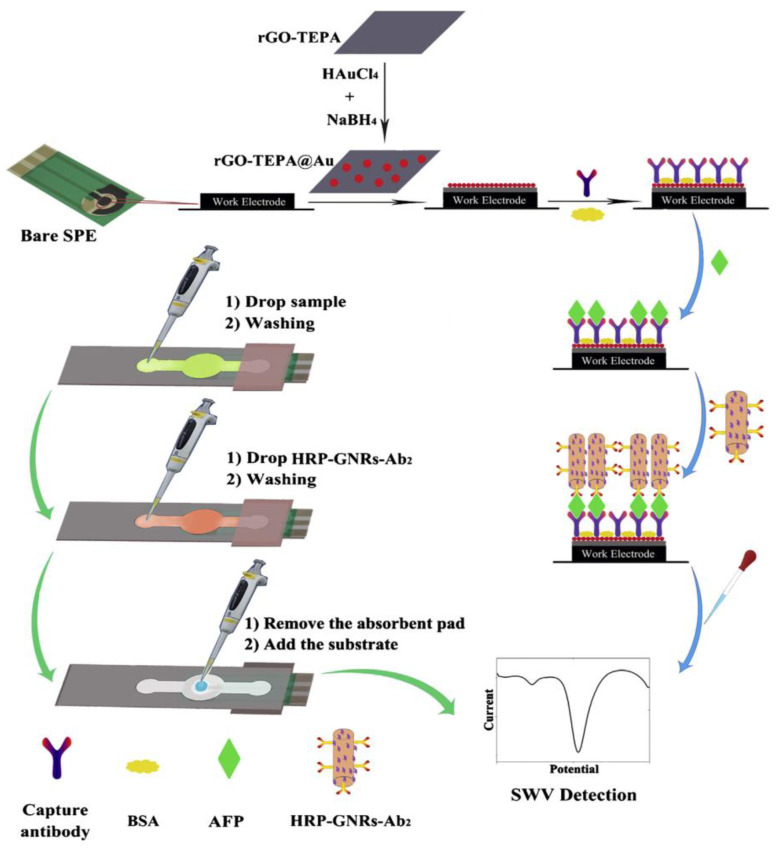
Schematic displays of the modification and assay procedure for the implementation of an immunosensor for the determination of alpha-fetoprotein (AFP) using paper-based microfluidic channels to integrate sampling, detection and adsorption zones, and a reduced graphene oxide (rGO)-tetraethylene pentamine (TEPA)/AuNPs nanocomposite for immobilization of specific AFP antibodies. Reproduced with permission of Elsevier [35].

The combination of SPEs with simple paper-based microfluidics (μ-PEI) exhibits several advantages for the preparation of electrochemical biosensors compared with conventional analytical devices fabricated with other substrates (glass, silicon, or polymers). The resulting devices are inexpensive, easy to fabricate, and compatible with a variety of chemical or biochemical applications [35]. Cellulose papers with high surface area have become useful substrates in combination with SPEs for prototyping new point of care testing devices (POCTs) involving microfluidic systems in clinical diagnostics. Among the recent designs, those involving immunoassays constitute a challenge where the stability of the immunoreagents and the preparation of a surface capable of promoting electronic transfer to effectively enhance the assay sensitivity and selectivity, are critical factors. An illustrative method is that reported for the determination of alpha-fetoprotein (AFP). The method used paper-based microfluidic channels to integrate sampling, detection, and adsorption zones, as well as an rGO-tetraethylene pentamine (TEPA)/AuNPs nanocomposite for immobilization of specific AFP antibodies and sensitive detection (Figure 5) [35]. AFP is one of the most important biomarkers in diagnosing hepatocellular carcinoma, and in the case of pregnant women, it is the first serologic biomarker to detect birth defects in a developing baby [36,37]. The immunoreaction was performed by applying the tested solution to the sample zone and letting it elute slowly to the detection zone where AFP was captured. Then, gold nanorods decorated with horseradish peroxidase and detector antibodies (HRP-GNRs-Ab_2_) were dropped onto the sample zone to form a sandwich-type configuration on the working electrode and SWV detection was carried out in the presence of H_2_O_2_. The calibration plot showed a wide linear range (0.01–100.0 ng mL^−1^) with a low LOD value of 0.005 ng mL^−1^.

Oher configurations involving label-free paper-based immunosensors with immobilized capture antibodies have been reported for the detection of hormones [40,42], CRP [43], interferon (IFN-γ) [45], cancer biomarkers [44,46], and cardiac biomarkers [41,47]. Among them, it is worth mentioning the platform described by Ruecha et al. [45] involving a paper-based microfluidic device coupled with a label-free electrochemical impedimetric immunosensor for the detection of IFN-γ in serum. This multifunctional cytokine, originally characterized by its viral activities, is primarily secreted by natural killer T cells as a part of the innate immune response to intracellular pathogens [48] and plays a crucial role related to inflammatory and antoimmune diseases. In Ruecha’s method, a wax-printing strategy was implemented to fabricate the paper electrode, which was screened with graphene modified ink to deposit polyaniline (PANI) and further covalent immobilization of the specific anti- IFN-γ antibodies. The increase of the charge transfer resistance with the cytokine loading provided a linear relationship with logarithmic concentrations of IFN-γ in the 5–1000 pg mL^−1^ range with a LOD of 3.4 pg mL^−1^.

**Figure 6:**
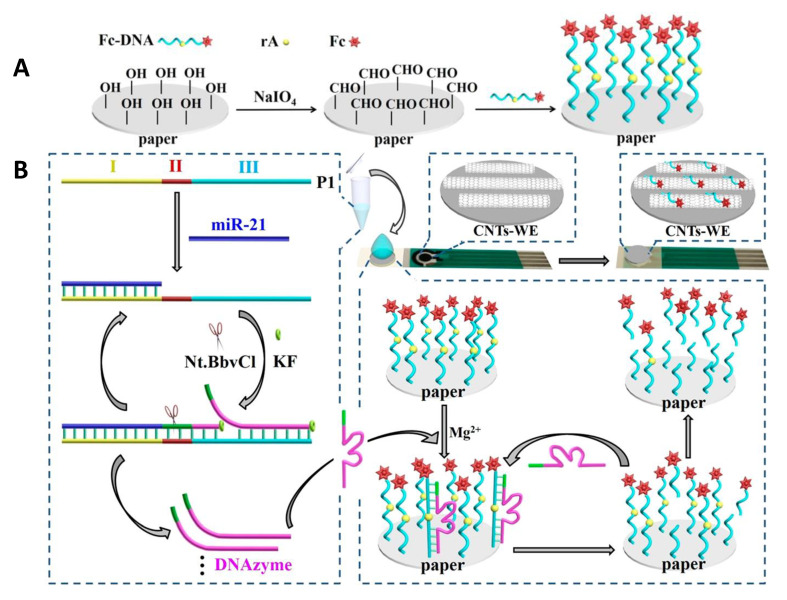
Schemes of (**A**) Ferrocene-labeled DNA (Fc-DNA) immobilization on paper and CNTs-SPEs, and (**B**) miRNA assay for the recognition of miR-21 (left), and the electrochemical response to the released DNAzymes (right). Reproduced and adapted with permission of American Chemical Society [38].

Although the number of paper-based electrochemical biosensing platforms has increased in the last few years, the vast majority of the reported methods involve the use of enzymes and antibodies as recognition elements, and so far, they have not been expanded to nucleic acid-based assays. In this context, Liu et al. [38] prepared a paper modified with signal molecule-labelled DNA and a screen-printed electrode along with target recognition solutions to achieve the detection of multiple biomarkers. The method is based on the target-induced synthesis of Mg^2+^- dependent DNAzyme for catalyzing the cleavage of substrate DNA from paper, taking advantage of the high specific target-triggered polymerization/nicking and DNAzyme-catalyzed signal amplification. The performance of this method was evaluated using a microRNA recognition probe for lung cancer-specific miR-21, a phosphorylated hairpin probe for targeting alkaline phosphatase (ALP), and a DNA aptamer for carcinoembryonic antigen (CEA). Ferrocene-labeled DNA (Fc-DNA) was immobilized on paper by functionalizing it with aldehyde groups and further Schiff-based reaction (Figure 6A). Then, the paper-electrochemical biosensor was prepared by sticking the Fc-DNA modified paper onto a plastic slide and carbon nanotubes modified SPE. As an example, the fundamentals of miR-21 detection are illustrated in Figure 6B. After incubation with the recognition solution that contains the ssDNA probe (P1), KF polymerase and nicking endonuclease Nt.BbvCI, the polymerization via KF activity is initiated to extend the 3’-end, providing dsDNA with recognition site for endonuclease whose activity to cut one strand of dsDNA generates new replication sites. Then, the Mg^2+^-dependent DNAzyme strand is displaced and released. This cycle produces a large amount of DNAzyme strands that fold into the catalytically active loop structure and bind to immobilized Fc-DNA resulting in the release of DNAzyme strands and cleaved Fc-shorter ssDNA from the paper, which diffuses to the surface of CNTs-SPE giving a DPV response.

Among paper-based electrochemical DNA sensors, configurations developed for the detection of human papillomavirus (HPV) [54] and human immunodeficiency virus (HIV) [55] are particularly relevant. Teengam et al. [54] reported a graphene-PANI modified electrode with immobilized anthraquinone-labeled pyrrolidinyl peptide nucleic acid probe (AQ-PNA) for the detection of a synthetic 14-base oligonucleotide target with the sequence of HPV type 16 DNA by electrochemical measurement of the AQ response by SWV. A linear range of 10–200 nM and a LOD value of 2.3 nM were obtained. The performance of this biosensor was tested with the detection of PCR-amplified DNA. On the other hand, Cinti et al. [55] developed a series of paper-based strips for the electrochemical detection of single and double stranded DNA, which were successfully applied to a synthetic PCR amplified dsDNA sequence related to HIV in serum samples. Paper-based AuNPs-SPE platforms and triplex forming oligonucleotides (TFO) including Methylene Blue (MB) were used as the recognizing probes. 

## 3. Wearable Printed Electrodes for Biosensing Applications

Due to the booming research activity in the field of wearable and/or flexible printed electrodes, excellent reviews have been recently reported highlighting the versatility and tremendous potential of these devices [61,62,63,64,65,66,67,68,69]. Therefore, this section is just limited to give a rough overview of late advances and prospects to draw the current landscape of wearable and flexible printed electrodes (not implantable) for biosensing. Accordingly, only a few of the most representative methods reported during 2018 and 2019, applied mainly to clinical diagnosis and environment monitoring, are critically discussed. Table 3 summarizes the main features of these methods.

**Figure 7:**
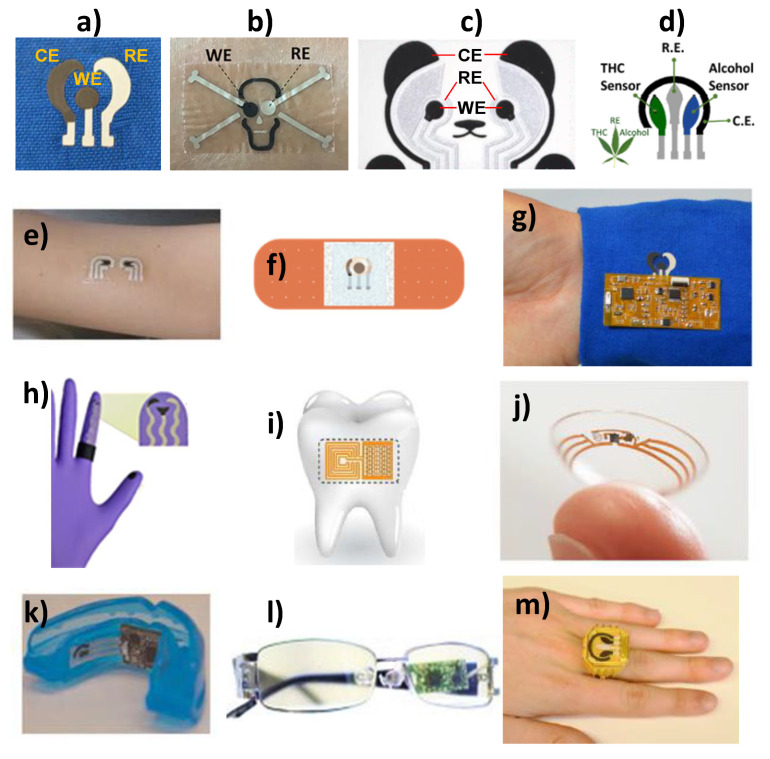
SPEs printed on flower (**a**), skull (**b**), panda bear (**c**), and marijuana (**d**) shapes. SPEs fabricated on a temporary tattoo (**e**), bendable bandage (**f**), textile substrate (**g**), glove (**h**), water-soluble silk thin-film substrates (transferred to tooth enamel) (**i**), contact lens (**j**), or incorporated in a mouthguard (**k**), eyeglasess (**l**) or ring (**m**). Reprinted and adapted with permission of Springer [68] (**e**,**j**,**k**) Wiley [81] (**f**), Elsevier [82] (**a**,**g**), Elsevier [82](**b**), Wiley [83] (**c**), Wiley [65] (**h**), Nature Research [84] (**i**), Elsevier [85] (**l**), and Elsevier [86] (**c**,**m**).

SPEs can be easily printed in a variety of shapes (flower, skull, marijuana, panda bear, etc., Figure 7a–d) and sizes and can be modified with different biological elements and nanomaterials. Leveraging on these advantages, screen printing has been employed to construct affordable wearable printed electrochemical sensors to provide real-time information on both the wearer’s health and performance (opening the door to individualized medicine) and the surrounding environment. In the physiological monitoring field, the active sensor surface is in close contact with the epidermis (oral mucosa in the mouth, stratum corneum or skin) to detect relevant biomarkers such as glucose [70] and ethanol [71] in different informative biofluids (saliva, sweat, tears). However, for monitoring the wearer´s environment, the sensor faces away from the epidermis in the direction of the surrounding to detect risk of exposure to chemicals [63]. Moreover, the recently explored robotic assisted strategy implies that the robot fingertips are kept in close contact with the target sample [72].

The spectacular growth witnessed in wearable printed sensors is largely due to the development of novel materials that imparted the resulting sensors the capabilities to fold, bend, stretch, and repair, ensuring their performance during on-body applications under extreme tensile stress [73]. Wearable electrochemical sensors have been implemented on head-to-toe wearable platforms and in connection to different biofluids, environments and analytes [64]. For the realization of wearable applications matching the non-planarity and mechanical properties of the human body, electrochemical sensors have been printed on temporary tattoo, bendable bandage, gloves, contact lens, water-soluble silk thin-film substrates (transferred to tooth enamel) or textile substrates (GORE-TEX and Neoprene) (Figure 7e–j) [63] or incorporated in mouthguards, eyeglasses, or rings (Figure 7k–m). The great progress experienced by electronics in terms of flexibility and miniaturization [65,74,75,76], in the development of effective methods for stimulating/controlling of non-invasive bio-fluids collection and the proliferation of smart-phones and connected devices, together with a growing consumer demand for health awareness, and the imperative need for doctors to obtain as much objective and quality data from their patients as possible, have been crucial aspects in the development of fully implementable wearable electrochemical devices and in opening up new avenues for body-integrated electronics previously unattainable [74]. However, powering is still the main Achilles’ heel of these devices and the size and weight of the power source may limit the wearability of the biodevices and hinder the wearer´s activity. Therefore, the rational integration of power sources with biosensors is a desperate requirement and additional efforts are required to develop anatomically compliant, miniaturized, stretchable and flexible power sources [64,77].

In general, there are three different modes of integrating wearable biosensors and power supplies: (1) an external circuit connection which is bulky and cumbrous; (2) a flexible substrate-based integration; and (3) all-in-one integration [78]. The last two strategies, made possible by advances in device designs and micro/nanofabrication technologies, are more widely used. The second strategy implies each component is relatively independent and can be considered a general integration strategy applicable to diverse sensing systems without having to worry about structural compatibility between components but difficult to allow the level of miniaturization required. The third strategy is effective for miniaturized designs in which all the components suffer from similar deformations simultaneously. However, the endurability difference among them should be minimized to guarantee the normal function of the respective component.

Currently, wearable biodevices are powered mainly by (i) safe high energy wearable batteries; (ii) energy conversion devices (piezoelectric and triboelectric nanogenerators, which harvest the mechanical energy in human motions, such as walking, breathing, and waving arms; solar cells, which harness light energy; thermoelectric supercapacitors; biofuel; and water-voltage cells); (iii) energy storage devices (mechanically flexible energy storage elements, mainly supercapacitor, and lithium-ion battery); (iv) hybrid power supplies combining energy conversion with energy storage; and (v) wireless energy transfer (wireless coils, like RF antennas). Significant progress have also been made in self-power and energy-efficient or even energy-free systems devices, fueled by the development of high-efficiency energy acquisition approach and ultra-low power consumption technique [68,69,78,79,80].

As a previous step to on-body measurements, Payne et al. made an exhaustive study to characterize the effects of five different salts in physiologically relevant concentration ranges on the performance of a printed, flexible, wearable biosensor involving lactate oxidase and tetrathiafulvalene for the amperometric detection of lactate in sweat [87].

**Figure 8:**
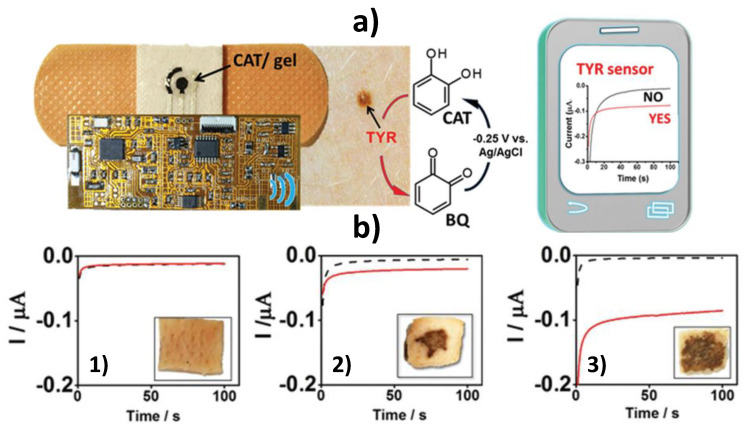
TYR biosensing using a bandage electrochemical sensor modified with a CAT-containing agarose gel and involving wireless chronoamperometric data transmission to a smart device (**a**). Chronoamperometric responses provided by the bandage sensors before (black line) and after (red line) 2 min interaction with skin pork samples untreated (1) and treated with 0.5 (2), and 2.5 mg mL^−1^ TYR (3) (**b**). Reprinted and adapted with permission of Wiley [81].

The extensive and pioneering work performed by Wang´s group in the development of wearable and flexible printed electrodes for biosensing in healthcare, food, and security fields should be noted. Wang’s team proposed the use of fully integrated wearable bendable bandage and minimally invasive microneedle-based sensors modified with catechol (CAT) for rapid and decentralized screening of skin melanoma through the amperometric detection of the benzoquinone (BQ) generated in the presence of the tyrosinase (TYR) biomarker (Figure 8). The bandage sensor exhibited high resiliency against mechanical strains due to the use of stress-enduring inks for its printing. These skin-worn sensors were interfaced to flexible electronic board that controlled the electrochemical operation and transmitted data wirelessly to a mobile device, and were used to screen biomarkers both on the skin surface (bandage sensor) or under the skin (microneedle device). They were applied to analyze TYR-containing agarose phantom gel and porcine skin [81]. These epidermal sensors allow skin cancer screening in less than 4 min obviating the need of painful solid biopsies and the associated delays and anxiety.

**Figure 9:**
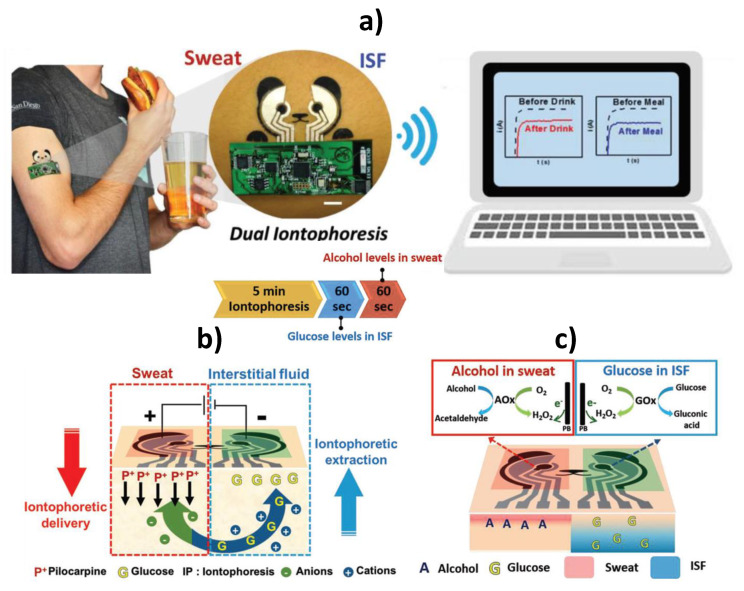
Wearable iontophoretic biosensing device developed on a printed tattoo platform for simultaneous glucose and alcohol monitoring in interstitial fluid (ISF) and sweat, respectively, and wireless real-time transmission of the recorded response (**a**). Schematic display of the iontophoretic operation to simultaneously induce generation of alcohol-containing sweat by iontophoretic delivery of pilocarpine at the anode and sampling of ISF glucose at the cathode by reverse iontophoretic (**b**); biosensing operations to detect amperometrically alcohol in the stimulated sweat and of glucose in the extracted ISF by measuring the hydrogen peroxide generated in the AOx and GOx enzymatic reactions (**c**). Reprinted and adapted with permission of Wiley [83].

The same group reported a strategy using a single wearable and flexible epidermal platform for the simultaneous yet independent noninvasive sampling and analysis of two different epidermal biofluids (sweat and skin interstitial fluid (ISF) with a blood-like composition) at two physically separate locations. This approach involves parallel operation of iontophoretic delivery of the sweat-inducing pilocarpine into the skin and reverse iontophoretic ISF extraction across the skin at anode and cathode, respectively (Figure 9a,b) [83]. The developed wearable device was implemented using a cost-effective screen-printing technique with body-compliant temporary tattoo materials for disposable single use and conformal wireless readout circuits, and integrated amperometric GOx and AOx biosensors (Figure 9c). It was used for real-time monitoring of alcohol and glucose levels in sweat and ISF, respectively, from individuals consuming food and alcoholic drinks.

**Figure 10:**
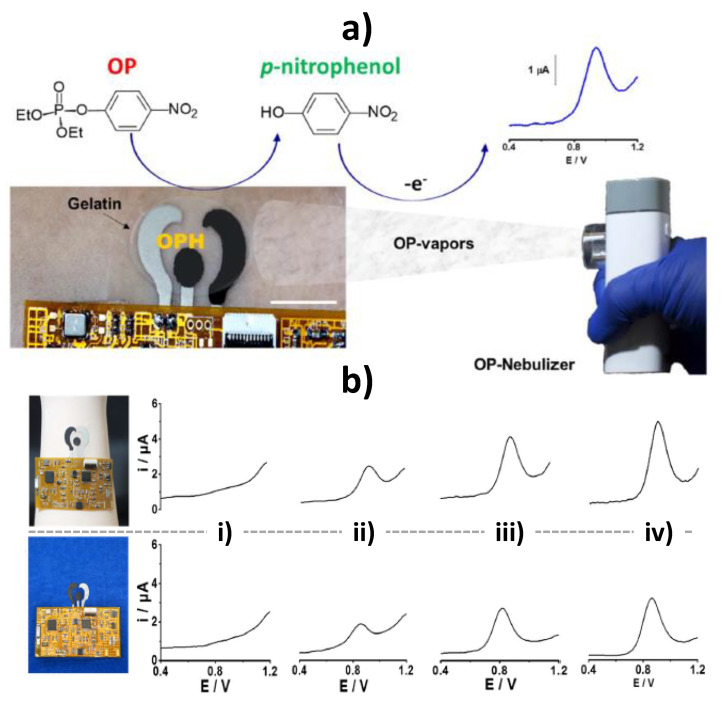
Epidermal tattoo organophosphorus hydrolase (OPH)-based biosensor for vapor-phase detection of OP through SWV measurements of the p-nitrophenol generated after interaction of the MPOx micro-droplets released from the nebulizer with the OPH layer (**a**). Pictures of the OPH based epidermal tattoo (up) and textile (down) biosensors integrated with the flexible electronic interface and SWV responses they provide upon spraying 0 (i), 5 (ii), 10 (iii), and 15 (iv) mM MPOx in the vapor phase (**b**). Reprinted and adapted with permission of Elsevier [82].

Flexible epidermal tattoo and textile-based electrochemical biosensors using stretchable organophosphorus hydrolase (OPH) enzyme electrodes have been developed for continuous vapor-phase detection of organophosphorus (OP) threats [82] (Figure 10a). These wearable sensors were fabricated with elastomeric inks and displayed resiliency toward mechanical stress expected from the wearer’s activity without compromising the biosensing performance. They were coupled with a fully integrated conformal flexible electronic interface providing square-wave voltammetry (SWV) detection of the enzymatically-generated nitrophenol product (Figure 10b) and wireless data transmission. The sensor achieved a LOD of 12 mg L^−1^ in terms of OP air density. The same group proposed also a wearable tattoo OPH–pH biosensor for real-time on-body potentiometric monitoring of G-type nerve agent simulants (using fluorine-containing OP nerve agent simulant diisopropyl fluorophosphate, DFP, as model) in both liquid and vapor phases. The OPH biocatalytic recognition phase was coupled on a flexible printed transducer with a pH-responsive poly(aniline) PANI layer for monitoring the proton release during the enzymatic hydrolysis of DFP by OPH. This skin-worn OP potentiometric sensor withstands severe mechanical strains without compromising the analytical performance and displays a wide dynamic range, fast response, high selectivity towards DFP and good reproducibility. These wearable OP biosensing devices hold considerable promise for real-time on-body detection and warning exposure to chemical threats such CWAs and pesticides in a variety of scenarios for triggering timely countermeasure actions, changing dramatically the protection of civilians, farmers, and military personnel.

**Figure 11:**
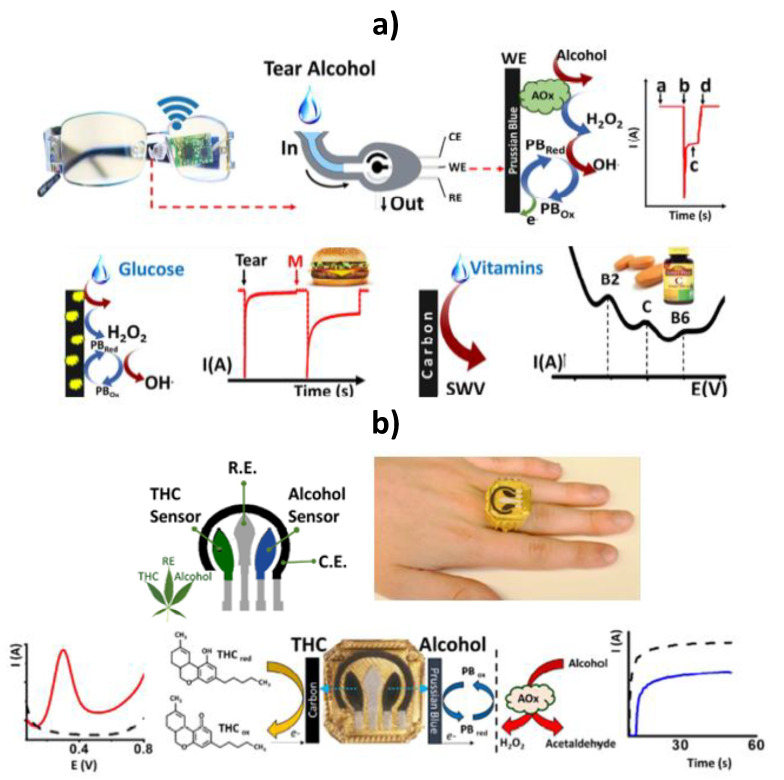
Schematic cartoon of the fluidic device, wireless electronics integrated into the eyeglasses platform, enzymatic detections of alcohol and glucose by chronoamperometry and non-enzymatic determination of vitamins by SWV in collected tears (**a**). Ring-based sensor platform embedded with marijuana designed sensor for detecting THC and alcohol in undiluted saliva samples using SWV and chronoamperometry (**b**). Reprinted and adapted with permission of Elsevier [85] (**a**) and [86] (**b**).

Wang’s team reported recently a wearable eyeglasses-based tear biosensing system for non-invasive monitoring of key biomarkers [85]. The wearable tear bioelectronic platform integrates a microfluidic electrochemical detector into an eyeglasses nose-bridge pad and the wireless electronic circuitry into the eyeglasses frame and yielded a fully portable, convenient-to-use fashionable sensing device placed outside the eye region addressing drawbacks of sensor systems involving direct contact with the eye as the contact lenses platform (Figure 11a). The concept was used for real-time non-invasive detection of alcohol, glucose and multiple vitamins in tears in connection with enzymatic (AOx and GOx) biosensing fluidic system (alcohol and glucose) and rapid voltammetric scanning (vitamins). This tear alcohol sensing strategy exhibited good correlation to concurrent blood alcohol concentration (BAC) in the monitoring of alcohol intake in individuals over multiple drinking courses.

A novel sensor ring concept, comprising a powerful wireless electronic board embedded into a ring platform and a readily replaceable printed dual-sensor electrode cap, was developed for the simultaneous, direct and rapid field detection of D9-tetrahydrocannabinol (THC) and alcohol in diluted saliva [86]. The ring sensing platform contained a voltammetric THC sensor based on a multi-walled carbon nanotubes MWCNTs/carbon electrode and an amperometric alcohol biosensor involving Prussian-blue (PB) mediator, coated with AOx/chitosan reagent layer on the ring cap (Figure 11b). The dual-analyte THC/alcohol ring sensor system showed no cross talk and high sensitivity (0.5 μM THC and 0.2 mM alcohol). THC and alcohol were determined simultaneously in the same diluted saliva sample within 3 min without any interference from the matrix. This new wearable THC/alcohol ring sensor, readily expanded to detecting other drugs of abuse, is very promising for rapid testing of suspected drivers and for alerting users to their own levels before driving.

**Figure 12:**
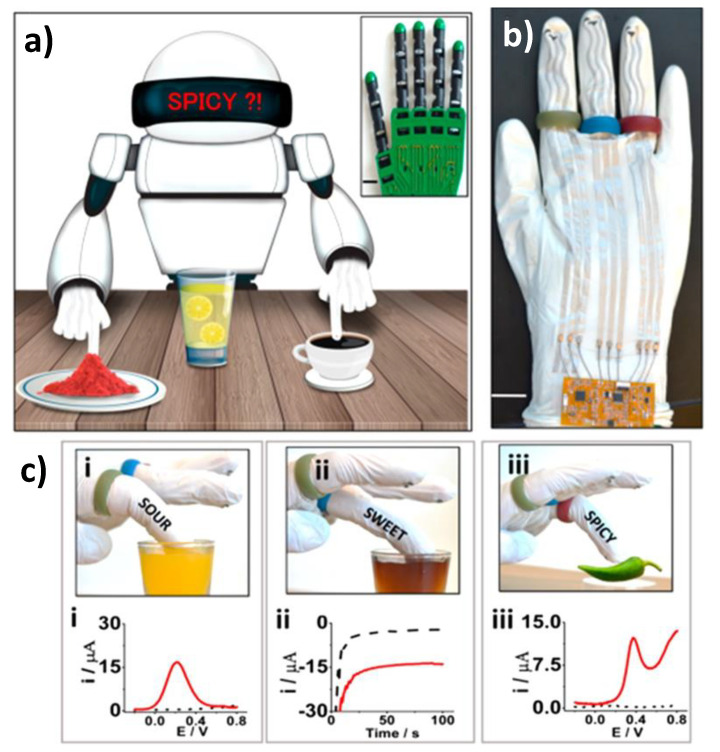
Automated taste discrimination in food samples through chemical sensing at the robot fingertips (**a**) Prototype of the screen-printed robotic sense fingers (carbon-printed sour-finger in green, GOx PB-printed sweet-finger in blue and carbon-printed spicy-finger in red) with long connections to the electronic interface (**b**). Images and corresponding electrochemical responses (in red) of: robotic sour-finger dipped in orange juice and the square wave voltammetry (SWV) signature of ascorbic acid (i), robotic sweet-finger in cherry juice and amperometry data of glucose (ii), spicy-finger on green-pepper, and SWV feedback response to the presence of capsaicin (iii). For comparison purposes the response obtained in phosphate buffer saline (PBS) response are displayed in black dotted lines (**c**). Reprinted and adapted with permission of American Chemical Society [72].

The same group have also reported in a pioneering way advances in wearable chemical sensor technology and flexible electronics to develop chemical sensing robotic fingers (printed on the robotic glove) for rapid discrimination between sweetness, sourness, and spiciness, via electrochemical monitoring of glucose, ascorbic acid, and capsaicin in different drinks (juices, sport and soft drinks and coffee) and extracts (green chili, red paprika and red pepper) (Figure 12) [72]. It is worth remarking that although it was out of the period to which this section has been restricted this group proposed also glove type wearable devices for use in forensic analysis (gunshot residues and nitroaromatic explosive compounds) [88]. This chemical sensing robotic skin is a key demonstration to spur future development of wearable printed electrodes, which offers great opportunity for automated chemical sensing machinery, facilitating robotic decision in a wide range of applications and even in potentially hazardous environments for human counterparts.

Wang’s group has reported very recently an epidermal AAOx biosensor able to monitor the dynamics of vitamin C in sweat after the intake of vitamin C pills and fruit juices [89]. This method combines the use of a flexible vitamin C tattoo patch fabricated on a polyurethane substrate with a localized iontophoretic sweat stimulation system. Chrono-amperometric cathodic detection of the oxygen cosubstrate consumption during the enzymatic reaction, demonstrates very interesting potential for personalized nutrition solutions. 

## 4. General Considerations, Challenges to Face, and Future Prospects

The advances that have occurred in recent years in screen-printing technology provide unimaginable possibilities in electrochemical sensing and biosensing. Paper-based and wearable (bio)sensors are two of the areas that benefit from such advances.

e-PADs show excellent opportunities for sensing and biosensing mainly in the environmental and clinical fields. For these purposes, different types of paper impregnated with the suitable reagents, modified with different nanomaterials (metal nanoparticles, carbon nanostructures, and nano-sized MIPs) either in the absence or in the presence of specific bioreceptors (enzymes, lectins, antibodies, and, to a much lesser extent, nucleic acids) have been proposed. The resulting devices exploit the interesting features offered by nanomaterials in terms of electrocatalytic properties, biocompatibility and high surface area, and the strong selectivity of biological molecules or MIPs, with the advantages of electrochemical detection. In the environmental field, e-PADs have been applied to the determination of heavy metals (Cu^2+^, Pb^2+^, Cd^2+^), anions (ClO_3_^−^, NO_2_^−^), gases (NO, NO_2_, DMMP), CWAs (mustard agent) microbial pathogens (*E. coli*), or other biotoxics (antibiotics or pesticides).

In the clinical field, e-PADs have been utilized for the determination of a wide variety of biomolecules including miRNAs, hormones (17β-estradiol, FSH), viruses (HPV, HIV), proteomic biomarkers of relevance in cancer and cardiovascular diseases (CRP, BNP, IFN-γ, CA125, cTnI, AFP, BChE activity, MMP9, and CEA), and other clinically relevant analytes (3-NT, glutathione, glucose, H_2_O_2_, serotonin, L-Tyr, Phe, acetylcholine and Cl^−^). Paper-based electrochemical (bio)sensors have been employed to determine target analytes in highly variable matrices—soils, exhaust gases, waters and industrial sewage, cellular extracted DNA, blood, plasma, serum, urine, sweat, exhaled breath, and pharmaceutical capsules. Remarkable achievements include the development of disposable gPADs and the combination of SPEs with simple paper-based microfluidics (μ-PEI) with great interest in clinical diagnostics for prototyping new POCTs.

Apart from the interesting features derived from the use of paper as substrate such as porosity, capillarity, high surface area/volume ratio, disposability, lightness, flexibility, eco-friendliness, and low-cost, the filtration properties of this particular substrate have also been exploited. Moreover, some of these (bio)devices exhibit antifouling properties that are highly pursued to ensure the proper functioning of the devices in real world matrices and their use to determine low levels of analytes directly in such matrices involving simple and straightforward protocols.

Focusing on printed wearable devices devoted to electrochemical biosensing, new generation of printed wearable devices include soft, biocompatible, stretchable, and anatomically compliant devices with multifunctional characteristics that enable efficient bio-integration and withstand high tensile stress associated with on-body applications. Over the past two years, their applications have been geared mostly toward healthcare and environmental fields. To date, they have demonstrated preliminary potential for individual or multiplexed electrochemical determination in or near real time of relevant analytes in the agro-food, clinical and environmental areas (ethanol, drugs of abuse, lactate, glucose, vitamins, TYR, OP nerve agents) in different matrixes such as biofluids (sweat, saliva, tears, and ISF), vapor phases, and skin. So far, printed temporary tattoo and bandage sensors and printed sensors mounted in mouthguards, eyeglasses, or rings have been proposed for healthcare applications. Regarding environmental applications sensors have been printed on textile and gloves. Particularly noteworthy is the use of this type of sensors to screen biomarkers related with cancer in skin, the pioneering coupling with a parallel iontophoretic mechanism (extraction and delivery) for simultaneous sampling of different biofluids at separate locations, the enclosing within a microfluidic chamber for continuous monitoring and the development of a wearable taste-sensing robotic technology to discriminate between different flavors in liquid and solid food samples and an epidermal enzymatic biosensor for noninvasive nutrition status assessments.

However, it should be noted that, at present, the remarkable capabilities for taking preventive intervention of health and environmental risks, have been proved using proof-of-concept prototypes and for a limited number of samples, analytes, and biosensing approaches. Therefore, a thorough validation with large population studies and coordination and collaboration with medical practitioners to correctly interpret the data, and a better understanding of the correlations between analyte concentrations in the blood and noninvasive biofluids are required to underpin clinical acceptance. In addition, their extension to bioaffinity assays, particularly challenging since they require complex, multistep and usually not reversible protocols, is highly demanded to make other important biomarkers (proteins, DNAs, and RNAs) accessible to monitoring. Indeed, despite the great customization potential of these printed wearable electrochemical devices, important efforts are required for advancing them from prototypes to field devices and for their widespread commercial exploitation. Additional efforts are required to improve durability and robustness of wearables´ batteries. Solutions are required to decrease the power consumption of devices for extended monitoring periods, which include battery consumption minimization, and developing of replaceable and flexible power source with continuous and long-time output or self-powering wearable devices.

It is evident that the development of wearable printed electrochemical devices, which so far has only scratched the surface of their tremendous potential, is poised to grow very rapidly over the next decade, bringing a considerable advance to the field of wearable devices. Although many challenges that impede the widespread adaptation of this field for commercial applications have been addressed due to the recent years endeavors in material science, microfluidics, nanotechnology, and biotechnology, together with the work in unison of researchers from diverse fields, there are still some outstanding issues before their full potential will be realized and exploited in our real lives. However, there is clearly room for them within many applications (forensic, food assessment, healthcare, security), where rapid screening and timely chemical information is critical.

The exciting new developments anticipated to come in the foreseeable future in both paper-based and wearable electrochemical (bio)sensors will certainly change and improve our daily lives providing eco-friendly, affordable and efficient solutions for smart healthcare (preventive medicine, precision medication and management of chronic diseases) and wellness moving the lab to our body (skin, mouth, and eyes), minimizing risks of exposure to chemical threats and drug impaired driving concerns. Taking into account the demands for ordinary users facing other issues such as the diversity of analytical targets in the practical applications and the selectivity, stability and recyclability of these screen-printing biodevices, additional future research can be predicted in this field. Moreover, continuous investment in material preparation and fabrication process perfection (including ingenious structural designs) and in achieving higher integration between the multifunction sensing units and auxiliary components (power supply, communication and even signal processing and displaying) will play a significant role in constructing cost-effective and consistent (bio)sensors, easily adopted by current society.

Indeed, today, it is timely to stress that the adaptation of mask filtration systems to include transducers for aerosol/viral detection, although very difficult, would represent a disruptive technology for the detection of pandemics such as the SARS-CoV-2 coronavirus we are experiencing.

## Figures and Tables

**Table 1:** Screen-printed paper electrochemical (bio)sensors for environmental applications.

Configuration	Technique and Method	Detection	Analyte/Sample	Analytical Characteristics	Ref.
Origami gas-sensing paper-based with CuNPs/SPGE	Gas absorption and electrocatalytic oxidation of NO_2_ reduced form in the presence of CuNPs	DPV (nitrite)	NOx/air, exhaust gases from cars	0.03 vppm	[14]
Origami paper based multiple biosensor with BChE, AP or Tyr	Detection of TCh, 1-naphthol or 1,2-benzoquinone	Differential amperometry	paraoxon, 2,4-DCPA, atrazine	L.R: 2–100 ppb LOD: 2 ppb	[21]
PANI/G/PEO/p(VB-co-VA-co-VAc) on cellulosic paper	Conductive paper with printed sensor patterns	Resistance changes	nerve gas (DMMP)	L.R.: 3–30,000 ppb LOD: 3 ppb	[15]
ChOx/PB/CBNPs/office paper SPE	Inhibition of ChOx activity	Amperometry (H_2_O_2_)	Sulphur mustard (Yprite)	L.R: 1–4 mM LOD: 0.9 mM	[11]
Microfluidic device with chromatographic paper/CE	BQ mediated *E. coli* respiration	Amperometry (HQ)	pesticides/soils, vegetables	LOD: 37.5 μg g^−1^ (triazolone)	[20]
G/AuNPs/mixed cellulose ester filter paper	Direct electrochemical oxidation	DPV (NO_2_^−^)	nitrite/waters	L.R: 0.3–720 μM LOD: 0.1 μM	[13]
CNTs/Chit/SDS/cellulosic paper with electrodeposited Bi	Anodic stripping previous accumulation at −1.2 V for 240 s	SWASV	Pb^2+^/waters	L.R: 10–200 ppb LOD: 6.74 ppb	[12]
G/CNTs/ionic liquid/cellulosic paper with electroplated Bi	Anodic stripping previous accumulation at −1.3 V for 300 s	SWASV	Cd^2+^, Pb^2+^/wood	L.R: 1–50 μg L^−1^ LOD: 0.2 μg L^−1^	[22]
[PMo_11_VO_40_]^5−^/Whatman #4 filter paper/SPE	Direct electrochemical reduction	CV	ClO_3_^−^/soil	L.R: 0.312–2.5 mg mL^−1^ LOD: 0.15 mg mL^−1^	[10]
CB/Prussian Blue paper-based SPE	Reagent-free nitrocellulose membrane with enzyme substrate BTCh	Differential amperometry	nerve agents (paraoxon)	L.R: up to 25 μg L^−1^ LOD: 3 μg L^−1^	[23]
CNFs or rGO/AuNPs	Whatman Grade 1 cellulose paper modified by ink (bottom side) and nanomaterials (upper side)	LSV after preconcentration at +0.2 V vs Ag for 600 s	Hg(II)/river waters	L.R: up to 1.2 μM LOD: 30 nM	[24]
SiNs/paper/rGO/SPCE	Paper-based immunocapture assay with anti-EE2	SWV	EE2/ waters	L.R: 0.5–120 ng L^−1^ LOD: 0.1 ng L^−1^	[25]
carbon black ink/filter paper SPE	Direct electrochemical oxidation	SWV	BPA/waters	L.R: 0.1–0.9; 1–50 μM LOD: 0.03 μM	[26]

AP: alkaline phosphatase; BChE: butyrylcholinesterase, CB: carbon black; Chit: chitosan; CFU: colony forming unit; ChOx: choline oxidase; CNF: carbon nanofibers; DCPA: 2,4-dichloro-phenoxyacetic acid; DMMP: dimethyl methylphosphomate; *E. coli*: *Escherichia coli*; EE2: ethinyl estradiol; EIS: electrochemical impedance spectroscopy; G: graphene; HQ: hydroquinone; LOD: limit of detection; L.R: linear range; CNTs: carbon nanotubes; PANI, polyaniline; PB, Prussian Blue; PEO, polyethylene oxide; p(VB-co-VA-co-VAc): poly(vinylbutyral-co-vinyl alcohol-co-vinyl acetate); rGO: reduced graphene oxide; SPE: screen-printed electrode; SPGE: screen-printed gold electrode; SDS: sodium docecylsulfate; SiNs: silica nanoparticles; SWASV: square-wave anodic stripping voltammetry.

**Table 2:** Screen-printed paper electrochemical (bio)sensors for clinical applications.

Configuration	Technique and Method	Detection	Analyte/Sample	AnalyticalCharacteristics	Ref.
Fe(CN)_6_^3−^/banana peel tissue/SN-MPTS/paper	L-Tyr oxidation catalyzed by tyrosinase and mediated by Fe(CN)_6_^3−^	DPV	L-Tyr/plasma	L.R: 0.05–600 μM LOD: 0.02 μM	[39]
MWCNTs/THI/AuNPs/SPE	Label-free microfluidic paper based immunosensor with immobilized anti-E2	DPV (THI)	17β-estradiol (E2)/serum	L.R: 0.01–100 ng mL^−1^ LOD: 10 pg mL^−1^	[40]
(NH_2_-G)/THI/AuNPs/SPE	Label-free microfluidic paper based immunosensor with immobilized anti-BNP	Amperometry	BNP/serum	L.R: 0.05–30 ng mL^−1^ LOD: 12 pg mL^−1^	[41]
rGO/THI/AuNPs/SPE	Label-free microfluidic paper based immunosensor with immobilized anti-FSH	DPV (THI)	FSH/serum	L.R: 1–100 mIU mL^−1^ LOD: 1 mIU mL^−1^	[42]
rGO-TEPA/AuNPs/SPE	Microfluidic paper-based immunosensor with immobilized anti-AFP; HRP-GNRs-dAb as signal probe	SWV (H_2_O_2_/OPD)	AFP/serum	L.R: 0.01–100 ng mL^−1^ LOD: 0.005 ng mL^−1^	[35]
L-Cys-AuNPs/G/SPE	Label-free origami paper based immunosensor with immobilized anti-CRP	EIS (Fe(CN)_6_^3−/4−^)	CRP/serum	L.R: 50–10^5^ ng mL^−1^ LOD: 15 ng mL^−1^	[43]
Q-MA/SPGE	Label-free microfluidic paper based immunosensor with immobilized anti-CEA	DPV	CEA/serum	L.R: 1–100 ng mL^−1^ LOD: 0.33 ng mL^−1^	[44]
DPA/Ag/G/SPCE	Microfluidic plastic-paper based immunosensor with immobilized anti-AFP	EIS	AFP	L.R: 1–10^4^ ng mL^−1^ LOD: 1 ng mL^−1^	[37]
OPANI/G/SPE	Label-free microfluidic paper based immunosensor with immobilized anti-IFN-γ	EIS (Fe(CN)_6_^3−/4−^)	IFN-γ/serum	L.R: 5–10^3^ pg mL^−1^ LOD: 3.4 pg mL^−1^	[45]
rGO/THI/AuNPs	Label-free microfluidic paper based immunosensor with immobilized anti-CA125	DPV (THI)	CA125/serum	L.R: 0.1–200 U mL^−1^ LOD: 0.01 U mL^−1^	[46]
cMWCNTs/cellulose paper/SPE	Label-free paper based immunosensor with immobilized anti-cTnI	EIS (Fe(CN)_6_^3−/4−^)	cTnI/serum	L.R: 0.05–50 ng mL^−1^ LOD: 0.05 ng mL^−1^	[47]
Fe_3_O_4_@AuNPs@SiO_2_ MIP /Whatman paper/CPE	3D-ePAD Direct oxidation	LSV	serotonin/capsules, urine	L.R: 0.01–1,000 mM LOD: 0.002 mM	[30]
C/Ag/paper/SPE	Label-free detection, current decrease	SWV	3-nitrotyrosine	L.R: 500 nM–1 mM LOD: 49.2 nM	[27]
Patterned waxed paper screen-printed with silver ink	Electrochemical oxidation in the presence of silver	CV	chloride/serum, sweat	L.R: up to 200 mM LOD: 1 mM	[49]
Prussian Blue/paper/SPEs	Differential current measurements	amperometry	H_2_O_2_/simulated exhaled breath	L.R: 5–320 μM LOD: —	[29]
Prussian Blue/C black/wax patterned wax filter paper	Thiol-disulfide exchange reaction	Amperometry +0.3 V	glutathione	L.R: up to 10 mM LOD: 60 μM	[28]
CoPc/G/IL/paper/SPCE	Non-enzymatic detection	Amperometry +0.7 V	glucose/serum, honey, wine	L.R: 0.01–1.3–5.0 mM LOD: 0.67 μM	[50]
AuNPs/porous paper/SPE	Non-enzymatic detection	CV	glucose	L.R: 0.01–5 mM LOD: 6 μM	[51]
ATCh/G/Ag/AgCl ink/wax printing paper	ATCh hydrolysis by AChE giving TCh directly oxidized	Amperometry/TCh	AChE	L.R: 0.1–15 U mL^−1^ LOD: 0.1 U mL^−1^	[34]
PheDH/paper/ERGO/SPCE	Phe hydrolysis by PheDH in the presence of NAD^+^	Amperometry/NADH	Phe/neonatal blood	L.R: 1–600 μM LOD: 0.2 μM	[33]
paper-based wax printing/CB/SPCE	BTCh as substrate of BChE	Amperometry/TCh/PB	BChE activity/serum	L.R: up to 12 IU /mL LOD: 0.5 IU/mL	[52]
MBs/paper microfluidic/SPCE	On-chip single-step magneto-immunoassay with cAb-MBs and poly-HRP-biotin-dAb	Amperometry/H_2_O_2_/TMB	MMP-9/plasma	L.R: 0.03–2 ng mL^−1^ LOD: 0.01 ng mL^−1^	[53]
AQ-PNA/G-PANI/paper/SPCE	PNA-DNA duplexes obstruct electron transfer from AQ label	SWV/AQ	HPV/DNA from SiHa cell line	L.R: 10–200 nM LOD: 2.3 nM	[54]
MB-tagged TFO/AuNPs/paper/SPCE	filter and copy papers compared for detection of ssDNA or dsDNA	SWV/MB	HIV/serum	L.R: 3–3,000 nM LOD: 3 nM ssDNA;	[55]
CuO/IL/ERGO/SPCE/PAD	CuO/IL delivered from a HP D300 digital dispenser	Amperometry	Creatinine/human serum	L.R: 0.01–2.0 μM LOD: 0.22 μM	[56]
GOx-rGO-TEPA/PB-paper/SPE	3D paper-based microfluidic SPE	Amperometry H_2_O_2_/PB	Glucose/human sweat, blood	L.R: 0.1–25 mM LOD: 25 μM	[57]
rGO/AuNPs-paper-SPE	Wax-patterning on filter paper Whatman No1; rGO prepared from GO and dopamine	SWV	uric acid/urine	L.R: 2.5–1,000 μM LOD: 0.74 μM	[58]
Wax printed amino-functional graphene (NG)/THI/AuNPs and PB/PEDOT/AuNPs/SPE PADs	Label-free aptasensors	DPV	CEA, NSE/serum	L.R: 0.01–500 ng mL^−1^ (CEA); 0.05–500 ng mL^−1^ (NSE); LOD: 2 pg mL^−1^ (CEA); 10 pg mL^−1^ (NSE)	[59]
Wax screen printing patterns on cellulose paper/Nafion/Chit/GOx/PB/SPE	3D paper-based microfluidic SPE	Amperometry H_2_O_2_/PB	glucose/sweat	L.R.: up to 1.9 mM LOD: 5 μM	[60]

ATCh: acetylthiocholine chloride; AF: alpha-fetoprotein; AP: alkaline phosphatase; AQ-PNA:anthraquinone-labeled pyrrolidinyl peptide nucleic acid; AuNPs: gold nanoparticles; BChE: butyrylcholinesterase, BNP: B-type natriuretic peptide; BTCh: butyrylthiocholine; cAb: capture antibody; CB: carbon black; CEA: carcinoembryonic antigen; Chit: chitosan; CNFs: carbon nanofibers; CoPc: cobalt phthalocyanine; CRP: C-reactive protein; dAb: detector antibody; DCPA: 2,4-dichlorophenoxyacetic acid; DPA: diphenylalanine; EIS: electrochemical impedance spectroscopy; ERGO: elecrochemically reduced graphene oxide; FSH: follicle stimulating hormone; G: graphene; GNR: gold nanorods; HIV: human immunodeficiency virus HPV: human papillomavirus; HRP: horseradish peroxidase; IL: ionic liquid; MAQ: mercapto-amine quinone-functionalized receptor; MB: methylene blue; MWCNT: multi-walled carbon nanotubes; NSE: neuronspecific enolase; OPD: o-phenylenediamine; PANI: polyaniline; PB: Prussian Blue; Phe: phenylalanine; Q: quinone; Q-MA: quinone-based mercapto amine; rGO: reduced graphene oxide; SN-MPTS: 3-mercaptopropyl trimethoxysilane functionalized silica nanoparticles; SPE: screen-printed electrode; SPGE: screen-printed gold electrode; TCh: thiocholine; TEPA: tetraethylene pentamine; TFO: triple forming oligonucleotides; THI: thionine; TMB: tetramethylbenzidine.

**Table 3:** Wearable and flexible printed electrodes reported during 2018–2020 for biosensing applications.

Type of Wearable Sensor	Methodology	Analyte	Detection Technique	LOD	Application, Samples and Assay Time	Ref.
Flexible, wearable lactate sweat sensor	Biosensors using LOx and TTF	Lactate	Chrono-amperometry	—	Detection in artificial sweat	[87]
Bendable bandage and microneedle based sensors	In the presence of the surface TYR biomarker, its catechol substrate, immobilized on the transducer surface is rapidly converted to benzoquinone	TYR (Melanome biomarker)	Chono-amperometry	—	Melanoma screening in skin and tissues/Tyr-containing agarose phantom gel and porcine skin in less than 4 min (2 min of incubation and 100 s for the measurement)	[81]
Tattoo-like flexible iontophoretic platform integrated with electrochemical biosensors	Parallel operation of reverse iontophoretic ISF extraction across the skin and iontophoretic delivery of the sweat-inducing pilocarpine into the skin at separate locations and GOx and AOx-based biosensors	Glucose and alcohol	Chrono-amperometry	—	Simultaneous and real-time determination of alcohol and glucose levels on demand localized sampled sweat and ISF biofluids	[83]
Flexible epidermal tattoo and textile-based electrochemical biosensors	OPH-based skin- and textile-worn biosensors for continuous vapor-phase detection of OP threats integrated with a soft, flexible, skin-conforming electronic interface	Vapor-phase detection of OP nerve agents.	SWV	12 mg L^−1^ in terms of OP air density	Continuous and real-time vapor-phase detection of MPOx	[82]
Tattoo paper biosensor	Epidermal OPH–pH biosensor printed onto a temporary tattoo paper coated with PANi (for monitoring the proton release during the enzymatic hydrolysis of DFP by OPH) and with a PVA-acrylamide hydrogel which ensures surface distribution of the target DFP vapors	DFP in both liquid and vapor phases	Potentiometry	—	Real-time detection of DFP in both liquid and vapor phases	[90]
Eyeglasses platform for biosensing in tears	Enclosing the electrochemical biosensor within a microfluidic chamber, with the supporting electronics embedded onto the eyeglasses’ inner frame	Ethanol, glucose and multiple vitamins (B_2_, C and B_6_)	Chronoamperometry (ethanol and glucose) SWV (vitamins)	—	Real-time detection of alcohol intake and glucose and vitamins in human subjects	[85]
Ring-based dual sensing platform	Wireless electronic board embedded into a ring platform, along with a printed dual-sensor electrode cap comprising a voltammetric THC sensor based on a MWCNTs/carbon electrode and an amperometric alcohol biosensor based on a Prussian-blue transducer, coated with *AOx*/chitosan reagent layer	THC and ethanol	SWV (THC) and chrono-amperometry (ethanol)	THC: 0.5 μM; alcohol: 0.2 mM	Simultaneous detection of THC and ethanol in undiluted saliva sample within 3 min	[86]
Finger devices printed on the robotic glove	Robotic assisted automated taste sweetness, sourness, and spiciness discrimination in food samples	Glucose, ascorbic acid, and capsaicin.	Chrono-amperometry (ethanol and glucose) SWV (vitamins)	—	Ascorbic acid in orange juice, cola, lemon juice, sports drink, and pineapple juice; Glucose in: apple cider, sugar-free sports drink, cola, sugar-free energy drink, and apple juice; Capsaicin in: green chili extract, coffee, red paprika extract, watermelon juice and red pepper extract	[72]
Flexible printable tattoo electrodes	Flexible AAOx enzymatic biosensing tattoo patch fabricated on a polyurethane substrate and combined with a localized iontophoretic sweat stimulation system	Ascorbic acid	Chronoamperometry (Oxygen cosustrate depletion)	—	Sweat from subjects taking varying amounts of commercial vitamin C pills or vitamin C-rich beverages	[89]

AAOx: ascorbate oxidase; AOx: alcohol oxidase; GOx: glucose oxidase; DFP: diisopropyl fluorophosphate; ISF: skin interstitial fluid; LOx: lactate oxidase; MPOx: methyl paraoxon; MWCNTs: multi-walled carbon nanotubes; OP: organophosphorus; OPH: organophosphorus hydrolase; PANi: polyaniline; PVA: polyvinyl alcohol; SWV: square wave voltammetry; TTF: tetrathiafulvalene; THC: D9-tetrahydrocannabinol; Tyr: tyrosinase.
